# Malaria baseline survey in four special regions of northern Myanmar near China: a cross-sectional study

**DOI:** 10.1186/1475-2875-13-302

**Published:** 2014-08-07

**Authors:** Ru-bo Wang, Jun Zhang, Qing-feng Zhang

**Affiliations:** 1 National Institute of Parasitic Diseases, Chinese Center for Disease Control and Prevention, Shanghai 200025, People’s Republic of China; 2 WHO Collaborating Centre for Malaria, Schistosomiasis and Filariasis, Shanghai 200025, People’s Republic of China; 3 Key Laboratory of Parasite and Vector Biology, Ministry of Health, Shanghai 200025, People’s Republic of China; 4 Yunnan Office of Health Poverty Action (HPA), Kunming 650041, People’s Republic of China

**Keywords:** Malaria, Prevalence, Health facility, Long-lasting insecticidal bed nets, Special regions, Myanmar

## Abstract

**Background:**

Epidemiological data in the border area of the northern Myanmar near China are either of little accuracy or sparse of information, due to the poor public health system in these areas, and malaria cases may be severely underestimated. This study aimed to investigate malaria prevalence and health facilities for malaria services, and to provide the baseline information for malaria control in these areas.

**Methods:**

A cluster, randomized, cross-sectional survey was conducted in four special regions of northern Myanmar, near China: 5,585 people were selected for a malaria prevalence survey and 1,618 households were selected for a mosquito net-owning survey. Meanwhile, a total of 97 health facilities were surveyed on their malaria services. The data were analysed and descriptive statistics were used.

**Results:**

A total of 761 people were found positive through microscopy test, including 290 people for *Plasmodium falciparum*, 460 for *Plasmodium vivax*, two for *Plasmodium malariae*, and nine for mixed infection. The average prevalence of malaria infection was 13.6% (95% CI: 12.7-14.6%). There were significant differences of prevalence of malaria infection among the different regions (P < 0.01); 38.1% (95% CI: 28.3-48.0%) of health facilities had malaria microscope examination service, and 35.1% (95% CI: 25.4-44.7%) of these had malaria treatment services, 23.7% (95% CI: 15.1-32.3%) had malaria outreach services. 28.3% (95% CI: 26.1-30.6%) of households owned one or more long-lasting insecticidal bed nets (LLINs).

**Conclusion:**

The prevalence of malaria infection was high in the four special regions of northern Myanmar, near China. Malaria services in health facilities in these areas were weak. ITNs/LLINs owning rate was also low. The cross-border cooperation mechanism should be further strengthened to share the epidemical data about malaria, support technical assistance, and conduct joint malaria control or elimination activities.

## Background

Malaria is one of the most serious parasitic diseases in Myanmar, with more malaria deaths than any other country in Southeast Asia. According to the World Health Organization’s (WHO) Malaria Report, malaria incidence was 10.8% in 2008 [1]. On the border of Myanmar, the high malaria burden population usually serves as a large reservoir of infection and likely constitutes a source of infection for neighbouring countries [2]. For example, malaria prevalence in Myanmar migrants in Thailand is up to 20 times that of Thai local residents [3]. In the border area of northern Myanmar, near China, malaria burden is particularly high and malaria outbreaks occur frequently. Malaria outbreaks in November 2003 in Special Region 1^st^ (Kokang), Shan State resulted in the death of more than 100 people in 30 villages and sporadic outbreaks in 14 villages in three counties, Yunnan Province, and China [4].

Epidemiological data are either inaccurate or sparse of information on the border regions, due to the poor public health system in the remote border area of Myanmar; the malaria burden may be severely underestimated [5]. Surveillance and reporting systems for malaria incidence are relatively very weak, and information concerning malaria is insufficient. It is important to analyse the malaria burden in these areas for malaria control programme planning.

In order to describe malaria prevalence and health facilities’ malaria services in the border area of northern Myanmar, near China, a cross-sectional study was conducted in four special regions (targeted for Global Fund malaria project) in Myanmar, including the Special Region 2^nd^ (KR2), Kachin State; Special Region 1^st^ (Kokang), Shan State; Special Region 2^nd^ (Wa), Shan State; and, Special Region 4^th^ (SR4), Shan State, which are considered the malaria-endemic regions.

## Methods

### Study population and sampling design

This cross-sectional study was conducted between January and March 2008, the dry season in Myanmar. Most of the local residents in the four special regions are ethnic minorities (Kachin, Wa, Kokang, Dai, Lahu, Bulang and Hani). Kachin ethnic minority is the main ethnic in KR2 region, Kokang ethnic minority in Kokang region, Wa ethnic minority in Wa region, and Dai ethnic minority in SR4. Due to the lack of official population statistics, the total population estimated 545,000 (about 67,000 in KR2, 129,000 in Kokang, 277,000 in Wa, and 72,000 in SR4).

There are a total of 46 districts/counties in the four special regions. The study population was obtained using multistage cluster sampling. Firstly, the villages were divided into three categories (representing high, middle and low malaria epidemics, based on the malaria data in recent years from 30 health facilities set up by Health Poverty Action) in every district/county. Secondly, one village was selected randomly from each category, and three villages were selected in every district/county. The proposed sample size was based on a balance of operational feasibility and costs. Everyone in selected village was invited to take part in the malaria prevalence study. The participation rate was >85%; some villagers refused participation and some villagers were not at home, these were excluded from the study. Finally, 5,585 people for malaria prevalence and 1,618 households for mosquito net-owning status were surveyed using this approach. Meanwhile, a total of 97 health facilities, excluding private clinics and drugstores, in the four special regions were surveyed on their malaria control services.

### Survey questionnaires

The drafts of questionnaires and their completion instructions were developed based on the local situation in the special regions. A meeting was held with the participation of a group of malaria experts and investigators. The contents of the questionnaire were reviewed and modified as necessary in the meeting. Three questionnaires were used for survey: registration for microscopic examination, insecticide-treated bed nets (ITNs/LLINs) questionnaire for households, and a malaria service questionnaire for the health facilities. Demographic information comprising code, village, district/county, and individual information on name, gender and age were included in the microscopic examination registration. The ITNs/LLINs questionnaire comprised household size and the number of ITN/LLINs. The malaria service questionnaire mainly included information on malaria diagnosis, treatment, reporting, and ITN/LLINs distribution at the health facility.

### Data collection and quality control

The survey was conducted by six teams composed of one team leader, three investigators (microscopist, interviewer, translator) and a driver. Before the survey, all the team leaders and investigators received one week’s combination training. All the data were gathered by the investigating teams. Experienced microscopists were selected for taking and examining blood smears. The smears were read by one experienced microscopist. Once the positive smear was found, another microscopist would recheck it. One thick smear and one thin smear were made for each person, and smears were stained with Giemsa.

The quality of survey was supervised and monitored daily by a team leader at regional focal points to ensure the quality and quantity of data collected. The questionnaire and checklist was reviewed. Meanwhile, for the malaria prevalence survey, 20% of negative smears and 10% of positive smears were randomly checked by expert microscopists. The coincidence rate of negative slides was 89% (969/1092) and that of positive slides was 100% (70/70). Using Epidata3.1 software, all data were typed into a designed database and rechecked step-by-step.

### Data analysis

The descriptive statistics and differences in distribution were evaluated using the Chi-square (χ^2^) test by SPSS version 17.0 and P <0.05 was considered statistically significant.

### Ethical considerations

The study was reviewed and approved by the Ethical Committee of National Institute of Parasitic Diseases (NIPD), Center for Disease Control and Prevention (CDC). Informed consent was obtained from each respondent before conducting survey. Moreover, personal data of respondents were coded and kept as strictly confidential information. If the positive slide was detected, individuals testing positive would be treated with anti-malarials. The individuals with *Plasmodium vivax* were treated by chloroquine/primaquine; the other infected individuals were treated by ACT.

## Results

### Characteristics of study population and health facility

In the malaria prevalence study, a total of 5,585 people (1,277 people from KR2, 1,400 from Kokang, 1,535 from Wa, and 1,373 from SR4) were surveyed. The number of people in the two older age groups (50-59, 60- ) was less than those of the other age groups. The ratio of male to female was 0.8 in the four special regions. The details from these questionnaires are presented in Table 1.

**Table 1:** Demographic characteristics of residents surveyed in four special regions (n = 5,585)

**Characteristic**	**KR2 (n = 1,277)**		**Kokang (n = 1,400)**		**Wa (n = 1,535)**		**SR4 (n = 1,373)**		**Total (n = 5,585)**	
	**n**	**%**	**n**	**%**	**n**	**%**	**n**	**%**	**n**	**%**
AGE										
0-5	300	23.49	229	16.36	174	11.34	145	10.56	848	15.18
6-9	174	13.63	185	13.21	152	9.90	158	11.51	669	11.98
10-19	158	12.37	283	20.21	248	16.16	203	14.79	892	15.97
20-29	173	13.55	190	13.57	298	19.41	187	13.62	848	15.18
30-39	160	12.53	159	11.36	275	17.92	223	16.24	817	14.63
40-49	138	10.81	146	10.43	189	12.31	220	16.02	693	12.41
50-59	101	7.91	124	8.86	115	7.49	152	11.07	492	8.81
60-	73	5.72	84	6.00	84	5.47	85	6.19	326	5.84
SEX										
Male	572	44.79	675	48.21	705	45.93	589	42.90	2,541	45.50
Female	705	55.21	725	51.79	830	54.07	784	57.10	3,044	54.50
Male/Female		0.81		0.93		0.85		0.75		0.83

A total of 1,618 households (332 households from KR2, 449 from Kokang, 366 from Wa, and 471 from SR4) were interviewed on ITNs/LLINs ownership. Data for malaria services (diagnosis, treatment, reporting, and ITN/LLINs distribution) were collected from 97 health facilities, which were set up by the local governments and international non-government organizations (NGOs), but excluded private clinics and drug shops in the four special regions.

### Prevalence of malaria infection

A total of 761 people were found positive through microscopy tests, including 290 people for *Plasmodium falciparum*, 460 for *Plasmodium vivax*, two for *Plasmodium malariae*, and nine for mixed infection (*P. falciparum/P. vivax)*. The average prevalence of malaria infection in the four special regions was 13.6% (95% CI: 12.7-14.6%). There were significant differences of prevalence of malaria infection among the different regions (χ^2^ = 55.64, P < 0.01). Compared to the other two regions (Kokang and SR4), the prevalence of malaria infection in KR2 and Wa were higher. The details are presented in Table 2.

**Table 2:** Prevalence of malaria infection in four special regions (n = 5,585)

**Region**	**N**	**Total**		***P. f***		***P. v***		***P. m***		**Mix**		***P. f: P. v***
		**Positive**	**%**	**Positive**	**%**	**Positive**	**%**	**Positive**	**%**	**Positive**	**%**	
KR2	1,277	218	17.07	122	9.55	89	6.97	0	0.00	7	0.55	1.4
Kokang	1,400	149	10.64	47	3.36	102	7.29	0	0.00	0	0.00	0.5
Wa	1,535	261	17.00	82	5.34	175	11.40	2	0.13	2	0.13	0.5
SR4	1,373	133	9.69	39	2.84	94	6.85	0	0.00	0	0.00	0.4
Total	5,585	761	13.63	290	5.19	460	8.20	2	0.04	9	0.16	0.6

Children 0 to 5 years old (prevalence 20.8%), children 6 to 9 years old (28.3%) and people 10 ~ 19 years old (prevalence 20.2%) had higher malaria infection than the other people (Table 3). Prevalence was slightly higher among males (*P. falciparum* 5.4%, *P. vivax* 8.7%) than females (*P. falciparum* 5.1%, *P. vivax* 7.8%). The average ratio of *P. falciparum/P. vivax* in four special regions was 0.6.

**Table 3:** Prevalence of malaria infection by age and sex

**Characteristic**	**N**	**p.f (+)**	**p.f (%)**	**p.v (+)**	**p.v (%)**	**p.o (+)**	**p.o (%)**	**mix (+)**	**mix (%)**	**Total**	**Total (%)**	**p.f:p.v**
AGE												
0 ~ 5	848	68	8.0	106	12.5	0	0.0	2	0.2	176	20.8	0.6
6 ~ 9	669	75	11.2	107	16.0	2	0.3	5	0.7	189	28.3	0.7
10 ~ 19	892	68	7.6	110	12.3	0	0.0	2	0.2	180	20.2	0.6
20 ~ 49	2358	69	2.9	116	4.9	0	0.0	0	0.0	185	7.8	0.6
50~	818	10	1.2	21	2.6	0	0.0	0	0.0	31	3.8	0.5
Total	5585	290	5.2	460	8.2	2	0.0	9	0.2	761	13.6	0.6
SEX												
Male	2541	136	5.4	222	8.7	2	0.1	6	0.2	366	14.4	0.6
Female	3044	154	5.1	238	7.8	0	0.0	3	0.1	395	13.0	0.6
Total	5585	290	5.2	460	8.2	2	0.0	9	0.2	761	13.6	0.6

### Malaria services in health facilities

In 97 health facilities of the four special regions, 38.1% (95% CI: 28.3-48.0%) of health facilities had malaria microscope examination service for fever patients, 35.1% (95% CI: 25.4-44.7%) of those had malaria treatment services for malaria cases, 23.7% (95% CI: 15.1-32.3%) of those had malaria outreach services (i e, mobile, health education). Meanwhile, 32.0% (95% CI: 22.5-41.4%) of health facilities could report malaria case information.

However, the survey results were different between the four special regions. The malaria services in KR2 were the best, while Wa were better than Kokang and SR4. The malaria information reporting mechanism had not been set up in Kokang and SR4 (Table 4).

**Table 4:** Malaria services in health facilities

**Regions**	**Health facilities**	**Percentage of health facilities which had malaria services? (%)**			
		**Microscope test**	**Case treatment**	**Outreach service**	**Case report system**
KR2	18	100	100	100	100
Kokang	11	36.36	9.09	0	0
Wa	22	59.09	40.91	22.73	59.09
SR4	46	10.87	13.04	0	0
Total	97	38.14	35.05	23.71	31.95

Through checks of the records of 97 health facilities in 2007, only 10,136 fever patients received microscopy examination, 570 RDT tests were performed for fever patients, 3,059 malaria case received treatment appropriately and 13,866 LLINs had been distributed (Table 5).

**Table 5:** Malaria control activities in 2007

**Regions**	**Fever patients received microscopy examination**	**Fever patients received RDT diagnosis**	**Malaria case received treatment appropriately**	**LLINs distributed**
KR2	6,576	44	1,966	0
Kokang	1,675	0	0	11,038
Wa	1,760	526	1,093	2,828
SR4	125	0	0	0
Total	10,136	570	3,059	13,866

### Household ITNs/LLINs ownership

There was not any mosquito net re-treated with insecticide in the four special regions. 28.3% (95% CI: 26.1-30.6%) of households owned one or more LLINs, and all LLINs were distributed by NGOs, such as Health Poverty Action (HPA), Care, and Japanese Cooperation Organization (JICA). There was a large variation among the special regions: the ITNs/LLINs ownership in Kokang was higher than KR2 and Wa, and zero in SR4 (Table 6). A lot of LLINs were distributed by NGOs after a malaria outbreak in November 2003, which caused higher ownership in Kokang.

**Table 6:** Ownership of ITNs/LLINs in four special regions

**Region**	**Household**	**Household owning LLINs**	**Owning rate (%)**
KR2	332	118	35.54
Kokang	449	301	67.04
Wa	366	39	10.66
SR4	471	0	0
Total	1,618	458	28.31

Note: There was not any mosquito net re-treated by KO tab or the other insecticide in four special regions; During 2005-2007, LLINs were distributed in KR2 by HPA and in Wa by CARE; In 2007, LLINs were distributed in Kokang by JICA and CARE.

## Discussion

### Health system

Border malaria is one of the obstacles in malaria control for many countries [6]. In four special regions of northern Myanmar, which were conflict or potential conflict areas, the local ethnic minorities are politically, economically, culturally, and geographically marginalized from the society of Myanmar [7], and therefore the health system of the four special regions is very weak. Only a few hospitals are set up in towns, and only few malaria control activities were conducted in the villages due to lack of investment and the complex relationship between the central and local government. Malaria control in the four special regions was still in the non-planning stages. The poor capacity on malaria in the health facilities led to weak malaria services in these areas. The malaria control network should be strengthened as a priority. There was a lack of experienced health workers and medical equipment, and the technical training for the local health workers on diagnosis, treatment and case reporting should be conducted to improve access to early and accurate diagnosis and treatment for malaria. Meanwhile, there were some international NGOs conducting malaria control activities, such as LLIN distribution, which may play an important role in the malaria control.

### Malaria prevalence

Information about malaria epidemiology in the four special regions is very sparse [8,9]. This survey provided some data on malaria prevalence in the four special regions, which showed malaria prevalence was high. *Plasmodium falciparum* and *P. vivax* are the major malaria infections, mixed species infections (*P. vivax/P. falciparum*) and *P. malariae* infections are very low. The prevalence among children was higher than adults in these special regions, which was consistent with studies in some areas of Cambodia, Laos [10,11]. There were a little difference between males and females for malaria prevalence, which differ from some surveys Southeast Asia. These studies show that prevalence of male adults was higher the other peoples due to forest related activities [12]. The risk factor and reason of sex distribution of asymptomatic malaria in the four special regions couldn’t be discovered, due to lack of behaviour data. In the future, the behaviour survey should be included the malaria survey.

The proportion of *P. vivax* to *P. falciparum* infection was higher than that of malaria cases recorded in hospitals. The prevalence of *P. vivax* infection may be underestimated. The ethnic minorities in these areas have little access to routine health care service and would like malaria treatment to be available from the retail sector [6,13], and the patients with *P. vivax* infection probably seek treatment less than patients with *P. falciparum*[10].

These special regions in northern Myanmar are near the Yunnan Province, China, whereas many malaria cases are probably imported from Myanmar [14,15]. Cross-border behaviour was considered as high risk for malaria control in the border countries [16]. The cross-border cooperation mechanism in the border area should be further strengthened to share the epidemiological data about malaria, support technical assistance and conduct joint malaria control or elimination activities [17-19].

### Interventions

LLIN is an effective preventative measure in many malaria-endemic countries [20,21]. LLINs owning rate of household in the four special regions was very low, more and more LLINs should be distributed freely to the local villagers to protect from malaria infection. During the household interviews, it was found that some families did not use the LLINs distributed three years ago. It is suggested that malaria health education should be carried out to promote the use of LLINs [22].

In four special regions, the comprehensive interventions should be provided, such as bed nets, anti-malarial drugs, and vector control and health education. And there the local health systems and surveillance capabilities should be strengthened.

### Limitations

There are mountainous in these special regions and universal survey is difficult to be conducted because of funds and human resources. For sample survey, little malaria data and lack of official population statistics were big challenges. The sample villages were selected based on the malaria data from 30 health facilities by one NGOs (HPA). Then sampling bias may come from the preliminary data.

The sensitivity of microscopy declines rapidly at low parasite concentrations typical of asymptomatic infections [23]. PCR method was much more sensitive than microscopy in detecting malaria infections [24]. So the result in four special regions may be under-estimate the prevalence of infection. Additional studies are needed to estimate the implications of malaria activities conducted by the private clinics and drug shops.

Since 2008, the civil political situation had been changed and armed conflict recurred in Myanmar. The artemisinin resistance programmes were supported to control resistant parasites in Great Mekong regions. The international assistance from Global Fund, the Bill & Melinda Gates Foundation had dramatically increased in Myanmar. The prevalence data in four special regions over six years ago, as the historical information, will contribute to assess the above change.

## Conclusion

The prevalence of malaria infection was high and malaria services in health facilities were weak in the four special regions of the northern Myanmar, near China. The cross-border cooperation mechanism should be further strengthened to share the epidemical data about malaria, support technical assistance, and conduct joint malaria control or elimination activities.

## Competing interests

The authors have declared that they have no competing interests. The funders had no role in study design, data collection and analysis, decision to publish, or preparation of the manuscript.

## Authors’ contributions

RBW and JZ conceived the study. RBW and JZ participated in study design and data collection. RBW and QFZ analysed the data. RBW wrote the manuscript. All authors read and approved the final manuscript.
